# Middlesex Hospital

**Published:** 1831-01

**Authors:** 


### MIDDLESEX HOSPITAL.

### *Compound Fracture of the Leg; with Amputation.*

Wm. Caton, ætat. fifty-five, but in appearance ten or fifteen years older, was thrown down in the street, on the 4th of November, when a cart-wheel passed over his left leg, producing a compound fracture of the tibia and fibula, three inches above the ankle-joint, the integuments being bruised and lacerated over both bones. This case came under Mr. Mayo's care six days afterwards, that is, upon the 10th. At this time there was no appearance of action about the wound, and the constitutional disturbance was very trifling. Three or four days more passed, when the tongue became slightly furred, and the patient complained of more restless and uneasy nights.

On the 15th, Mr. Mayo carefully re-examined the state of the injured limb. Very little action seemed to be taking place about the fracture; yet the soft parts appeared to have been completely crushed, the bones to have been comminuted; the foot moved loosely at the fracture, as if it were attached by little more than the lacerated integuments and the vessels. The preceding night had been passed with great restlessness.

Under these circumstances, it was Mr. Mayo's impression that no salutary action would take place in the part, and that the patient would shortly experience symptoms of nervous irritation, or that some other equally serious consequence would ensue; and he determined, after consulting with his colleague, to remove the limb without loss of time.

The patient bore the operation extremely well, and had some refreshing sleep the ensuing night, the first, as he stated, which he had passed with comfort since his admission into the hospital. The stump is now nearly healed; but two circumstances, of an unfavorable nature, occurred, one in the operation, the other afterwards, which tend to evince the propriety of amputating in this case. The first is, that the anterior and posterior tibial arteries were found to be partly ossified, when the ligatures were applied to them. The second, that no adhesion took place in the stump for many days; and that, when at length some action had supervened, and the wound suppurated, the integuments, for an irregular surface, equal to about three inches square, sloughed.

---

*Compound Fracture of the Leg, followed by Tetanus.*

Daniel Power, a labouring man, ætat. forty, on the 4th of November, fell the height of a few feet, whereby the tibia and fibula of the right leg were broken, three inches above the ankle-joint, and the integuments covering the fractured part of the fibula were bruised and cut, but to no great extent. The surgeon, who dressed the wound of the integuments, doubted whether it communicated with the fracture. The patient, a few hours after meeting with this accident, was admitted into the Middlesex Hospital, the limb laid in junks, a spirit lotion applied over it, and every usual precaution taken.

The progress of the case promised to be favorable: the wound of the integuments did not, indeed, unite by adhesion, but it suppurated and granulated wholesomely: very little tumefaction of the limb occurred. A small dose of calomel appears to have been administered to this patient every night till the 10th of November, when he came under Mr. Mayo's care.

On the morning of the 10th, he complained of some difficulty in opening his mouth; but, as the gums appeared red and tumid, and the breath had a slight mercurial fetor, and as the mouth admitted of being opened to a considerable extent, and the temporal and masseter muscles felt soft and relaxed, and as the countenance was remarkably tranquil and composed, and the pulse not more than eighty, Mr. Mayo was inclined to view the symptom described as the effect of soreness of the mouth, from the calomel which had been taken. The bowels were freely opened.

But a few hours set the nature of the case beyond all doubt: during the night, the jaw became more locked, there was occasional difficulty in swallowing, and the muscles of the back of the neck were set and rigid. Calomel, in three-grain doses, with a grain of opium, was now administered every two hours.

At four o'clock P.M. on the 11th, no alteration having taken place, Mr. Mayo passed four acupuncturation needles into the rigid muscles of the neck: they were left inserted during half an hour, with no result. A silver catheter was likewise passed into the bladder, and retained there.

The next morning, Friday the 12th, the mouth had become distinctly affected with mercury: all the symptoms, however, of the complaint were aggravated; the patient was unable to swallow; the teeth could be separated to the smallest distance only, and the spasm had extended to the muscles of the back and abdomen; but its character was changed; the permanent spasm was not much more than the preceding day; but frequently, and at irregular intervals, the muscles of the back and abdomen became suddenly rigid in clonic spasm, and continued so affected for several seconds.

At one P.M. half an ounce of laudanum was administered in an injection; at the same time warm spirits of turpentine were applied to the wound, the appearance of which was healthy. At four o'clock the injection of laudanum was repeated. The patient continued to get worse, and died the same evening.

Upon examining the body, no unusual vascularity, or accumulation of water, was found upon the brain or medulla oblongata. A small gelatinous substance, not of recent formation, adhered to the dura mater, on the basilar process of the occipital bone.

On examining the fracture, the deep branch of the peroneal nerve appeared strained over the broken fibula, and bloodshot, but not more so than the adjacent parts. The patient had complained of no unusual pain in the fracture, nor had there been any spasm of the injured leg.

In a case of acute traumatic tetanus, treated by Mr. Mayo, the details of which are in a former Number of this Journal, the patient, who recovered, began to mend on the mouth becoming touched with mercury.

---

### *Removal of a Cicatrix.*

Mary Barnes, ætat. twenty-one, was admitted into the hospital, October 26th. Fourteen years ago, her clothes took fire, and she was grievously burnt on the neck, and chest, and right arm: it was two years before the wounds were healed. Many unsightly cicatrices ensued; the most inconvenient of which was a long seam, which extended obliquely in front of the elbow, and deprived the joint of one third of its capability of extension. On the 29th of October, Mr. Mayo dissected off a strip of this seam, about five inches in length, and two inches in breadth, and made two incisions transversely through the sound integuments, of sufficient length and depth to allow the elbow to be completely extended: in doing this, a large vein was divided. Oiled lint was laid on the wound, and the arm swathed in a bandage, and bound to a long splint, upon which it was retained, extended and supinated.

Nothing unfavorable has occurred in the progress of the case. The wound is now healed, and the elbow continues perfectly capable of extension. The splint is directed to be applied at night for a length of time to come.

---

### *Fits relieved by the Application of Moxa.*

George Russell, ætat. fifteen, has been an out-patient of the Middlesex Hospital, under Mr. Mayo's care, since the 26th of July. Eight years ago, when swinging upon a rope, he fell to the ground, and struck his head, and lay stunned for a short time. Three years after this, he was present when his brother fell over a bridge; upon which he was seized with strong and sudden giddiness. From this time he became liable to frequent seizures, which, when he became a patient of the hospital, had attained the following character:—

Six or seven times each day, he would, to use his own language, lose himself; not falling, but remaining perfectly motionless; in half a minute to three minutes, he would recover. Occasionally, once a week perhaps, the seizure was more severe, and he would drop perfectly senseless. He was liable to pains in the head, near the temples, which attacked either side indifferently. With this, his bowels acted regularly, and he appeared in other respects in good health. These attacks never supervened at night; and in the daytime, if he could keep perfectly quiet, he was free from them; but any thing that excited him, and especially any cause which led him to fix his attention steadily upon any subject, brought on a seizure.

Mr. Mayo desired that the moxa should be applied behind the ear, or to the back of the neck, every three days. After this remedy had been tried once or twice, the boy found himself materially better; and he has since attended regularly, (having found that, if he omits undergoing this treatment, he suffers by his neglect,) every three or four days, for the purpose of having the moxa applied. He has taken no medicine; for the last month, his diet has been restricted to vegetable food.

He is materially better, and continues to improve. It is some time since he has had one of the severer fits, and the slighter seizures are of rare occurrence. He is able to read, and to give his attention now to different pursuits. When he feels the fit coming on, he is able, by rushing into the air, and running some distance, to repel it: under these circumstances, as he begins to run, he feels inclined to turn round towards the left, the eyes rolling upwards and to the left, with a strong sensation of vertigo.

